# Ndst1, a heparan sulfate modification enzyme, regulates neuroectodermal patterning by enhancing Wnt signaling in *Xenopus*

**DOI:** 10.1111/dgd.12843

**Published:** 2023-03-03

**Authors:** Takayoshi Yamamoto, Yuta Kambayashi, Kohei Tsukano, Tatsuo Michiue

**Affiliations:** ^1^ Department of Life Sciences, Graduate School of Arts and Sciences The University of Tokyo Tokyo Japan; ^2^ Department of Biological Sciences, Graduate School of Science The University of Tokyo Tokyo Japan

**Keywords:** ectoderm, heparan sulfate, morphogen, Wnt, *Xenopus*

## Abstract

Neural tissue is derived from three precursor regions: neural plate, neural crest, and preplacodal ectoderm. These regions are determined by morphogen‐mediated signaling. Morphogen distribution is generally regulated by binding to an extracellular matrix component, heparan sulfate (HS) proteoglycan. HS is modified by many enzymes, such as N‐deacetyl sulfotransferase 1 (Ndst1), which is highly expressed in early development. However, functions of HS modifications in ectodermal patterning are largely unknown. In this study, we analyzed the role of Ndst1 using *Xenopus* embryos. We found that *ndst1* was expressed in anterior neural plate and the trigeminal region at the neurula stage. *ndst1* overexpression expanded the neural crest (NC) region, whereas translational inhibition reduced not only the trigeminal region, but also the adjacent NC region, especially the anterior part. At a later stage, *ndst1* knocked‐down embryos showed defects in cranial ganglion formation. We also found that Ndst1 activates Wnt signaling pathway at the neurula stage. Taken together, our results suggest that N‐sulfonated HS accumulates Wnt ligand and activates Wnt signaling in *ndst1*‐expressing cells, but that it inhibits signaling in non‐*ndst1*‐expressing cells, leading to proper neuroectodermal patterning.

## Graphical abstract

*ndst1* is expressed in anterior neural plate and the trigeminal region in *Xenopus* neurula. It enhances Wnt signaling in a region‐specific manner by accumulating Wnt ligand. This makes a clear boundary of Wnt signaling, and governs neuroectodermal patterning.

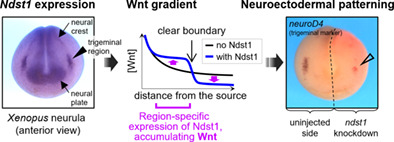

## 1 INTRODUCTION

Neural tissue comprises many types of cells that are derived from neural plate (NP), neural crest (NC), or preplacodal ectoderm (PPE) (Grocott et al., 2012; Thawani & Groves, 2020). These differentiation processes are regulated by multiple morphogens, such as Wnt, BMP, and FGF (Ozair et al., 2013; Rogers et al., 2009; Schlosser, 2014; Stern, 2006; Tsukano et al., 2022; Yamamoto et al., 2023). However, it remains unclear how these processes are precisely regulated by diffusing molecules.

Extracellular molecules are important regulators of morphogen distribution (Rogers & Schier, 2011; Yamamoto et al., 2022). Among them, an extracellular matrix component, heparan sulfate (HS), binds to morphogens, and restricts their diffusion (Yan & Lin, 2009). For instance, in neuroectodermal patterning, binding of a secreted BMP antagonist (gremlin) to HS is required for NC specification (Pegge et al., 2020).

HS is a glycosaminoglycan (GAG), which is covalently attached to a core protein. HS is modified in stepwise processes by multiple enzymes, initially by N‐deacetylase/N‐sulfotransferase (NDST), forming many different modification domains. Different modifications are thought to have discrete functions, that is, binding of specific morphogens, but the details have not been fully determined. In *Xenopus* gastrula embryos and *HeLa* cells, two modifications of HS mediated by NDST, N‐acetyl and N‐sulfo HS, create different domains on the cell membrane, and have different roles in Wnt/β‐catenin signaling pathway (Mii et al., 2017) (defined as Wnt signaling hereafter). Wnt8 is preferentially localized on N‐sulfo‐rich HS, whereas the antagonist, Frzb, is localized on N‐acetyl‐rich HS; however, the functions of Ndst are largely unknown in early development.

Wnt signaling is a key pathway during early embryogenesis, including NC specification (García‐Castro et al., 2002; Gomez et al., 2019; Ikeya et al., 1997; LaBonne & Bronner‐Fraser, 1998; Saint‐Jeannet et al., 1997). The ligand is secreted from the source, diffuses in the extracellular space, and binds to extracellular components, such as the receptor, Frizzled, and HS. In the Wnt/β‐catenin pathway, interaction of Wnt with Frizzled directs stabilization of β‐catenin through inhibition of GSK3β‐mediated phosphorylation, thereby promoting its nuclear localization. In ectodermal patterning, GSK3β overexpression disrupts NC specification (Saint‐Jeannet et al., 1997). Consistently, Wnt8 is essential for Fgf8a‐mediated NC induction ( Hong et al., 2008 ). At later stages, Wnt1 and Wnt3a, secreted from the dorsal neural tube, are required for NC differentiation (Ikeya et al., 1997; Saint‐Jeannet et al., 1997). In contrast, for specification of PPE, which is a neighboring tissue of NC, inhibition of Wnt signaling is required (Brugmann et al., 2004; Litsiou et al., 2005). This precise tuning of Wnt ligand distribution is thought to be essential for neuroectodermal patterning; however, it is not clear whether HS modifications are involved.

Among HS modification enzymes, NDST is the most well‐known for regulation of Wnt signaling (Mii et al., 2017). There are four *NDST* genes (*NDST1‐4*) in most mammals (Aikawa et al., 2001). These are also conserved in *Xenopus laevis*, and among them, *ndst1* shows high expression in early development (Michiue et al., 2017). Although *Ndst1* mutant mice showed defects of the forebrain and forebrain‐derived structures (Grobe et al., 2005), its function, especially in early neural development, is largely unknown. Here, we analyzed the role of Ndst1 to understand the mechanism of neuroectodermal patterning by utilizing *Xenopus laevis* embryos.

## 2 MATERIALS AND METHODS

### 2.1 *Xenopus* embryo manipulation and microinjection

All animal experiments were approved by the Office for Life Science Research Ethics and Safety at the University of Tokyo. Manipulation of *Xenopus* embryos and microinjection experiments were carried out according to standard methods, as previously described (Sive et al., 2000). Briefly, unfertilized eggs were obtained from female frogs injected with gonadotropin and artificially fertilized with testis homogenate. Fertilized eggs were de‐jellied with 4.6% L‐cysteine‐hydrochloride solution (pH 7.8), and incubated in 1/10× Steinberg's solution at 16–20°C. Embryos were staged as reported (Nieuwkoop & Faber, 1967). Amounts of injected mRNAs are described in the figure legends.

### 2.2 Injected samples

mRNAs were transcribed in vitro using an mMessage mMachine SP6 kit (Thermo Fisher Scientific). *ndst1* template was the same as in our previous paper (Mii et al., 2017).

Because *X. laevis* is an allotetraploid, we used a 1:1 mixture of two morpholino antisense oligonucleotides (MOs) targeting transcripts from both homeologs of *ndst1*: AGGAGTGGCACAAGCTCACAAATGC (*ndst1.L*) and AGGAATGGCACAAGCTCACAAATGC (*ndst1.S*) (Mii et al., 2017). Dextran tetramethylrhodamine (TMR) was used as a tracer (Invitrogen, D1818). Injected amounts of mRNAs and MOs are shown in the legends.

### 2.3 Whole mount in situ hybridization (WISH)

WISH was performed based on *Xenopus* standard methods (Harland, 1991) with slight modifications in the duration of washes and a hybridization temperature of 65°C. Plasmids for RNA probes were linearized and transcribed in vitro using a DIG RNA labeling mix (Roche). Enzymes for RNA probe synthesis are listed below.

### 2.4 β‐Catenin immunohistochemistry and confocal imaging

*Xenopus* gastrula embryos were fixed with MEMFA (0.1 M MOPS, pH 7.4, 2 mM EGTA, 1 mM MgSO_4_, 3.7% formaldehyde). Specimens were stained with β‐catenin antibody (C2206, Sigma, 1:1000). The specimens were observed using a confocal microscope (FV‐1200, Olympus).

### 2.5 RNA probe synthesis for in situ hybridization

**Table:** 

Gene	Restriction enzyme	Transcription enzyme	References
*ndst1*	EcoRI	T7	(Mii et al., 2017)
*sox3*	EcoRI	T3	(Nitta et al., 2006)
*foxd3*	EcoRI	T7	(Matsukawa et al., 2015)
*slug*	SpeI	T7	(Matsukawa et al., 2015)
*neurod4*	PstI	T7	(Matsukawa et al., 2015)
*islet1*	EcoRI	T7	(Matsukawa et al., 2015)
*krt12.4*	BamHI	T7	(Watanabe et al., 2018)

### 2.6 Phylogenetic analysis of *ndst* gene

Coding sequences of putative homologs of *ndst1.L* of *X. laevis* were collected from genes in cyclostomes, vertebrates, and echinoderms using ORTHOSCOPE v1.5.2 (Inoue & Satoh, 2019). Multiple alignments were constructed using MUSCLE program (Edgar et al., 2004). Phylogenetic trees were generated using the maximum likelihood method (ML) using MEGA11: Molecular Evolutionary Genetics Analysis version 11 (Tamura et al., 2021).

## 3 RESULTS AND DISCUSSION

**FIGURE 1:**
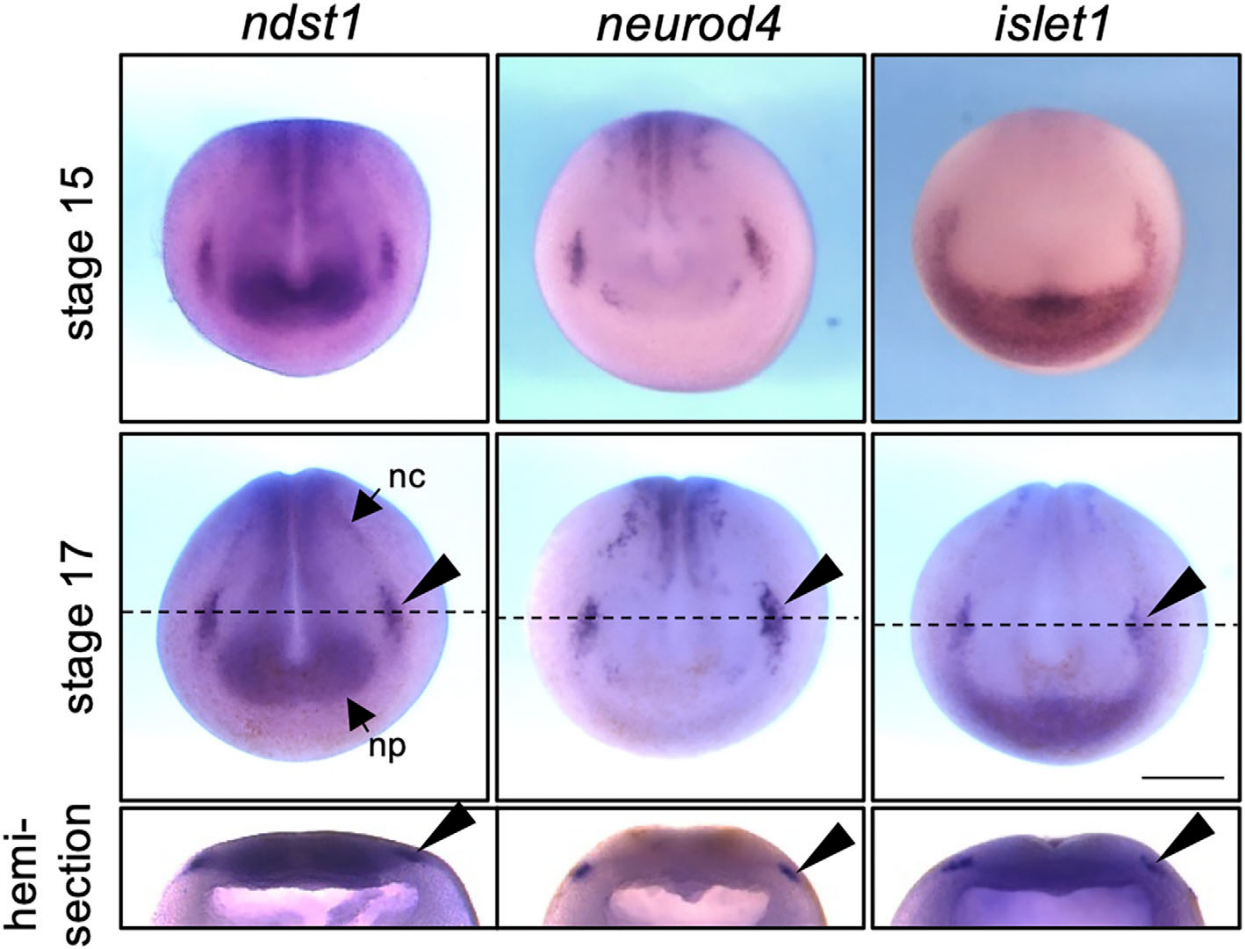
Expression pattern of *ndst1*, *neurod4*, and *islet1* in *Xenopus* neurula. Spatial expression pattern of *ndst1*, *neurod4*, and *islet1* was visualized by in situ hybridization at the neurula stage (stages 15, 17; anterior view, dorsal to the top). The specimen was cut with a razor at the dashed line (hemi‐section). All three genes were expressed at the lateral sensorial layer of neuroectoderm (arrowhead). Scale bar = 500 μm. nc, neural crest; np, neural plate.

*ndst1* is broadly expressed at the gastrula stage in *Xenopus*, but slightly more in the dorsal region (Mii et al., 2017). Its expression pattern at later stages was previously unknown. To investigate this, we carried out in situ hybridization, and found that at the early neurula stage (stage 13), *ndst1* is expressed around the anterior region (Figure S1). At later stages (stages 15, 17), *ndst1* is mainly expressed in anterior NP and a region lateral to it (Figure 1). This expression in the lateral region appeared to coincide with the expression pattern of a trigeminal marker gene, *neurod4* (*ath3*) (Hardwick & Philpott, 2015; Matsukawa et al., 2015). To investigate their expression patterns side‐by‐side, we performed in situ hybridization against trigeminal marker genes, *neurod4*, and *islet1*. Hemi‐sections show that these three genes are similarly expressed in the lateral region of the sensorial layer of epidermal ectoderm (Figure 1).

**FIGURE 2:**
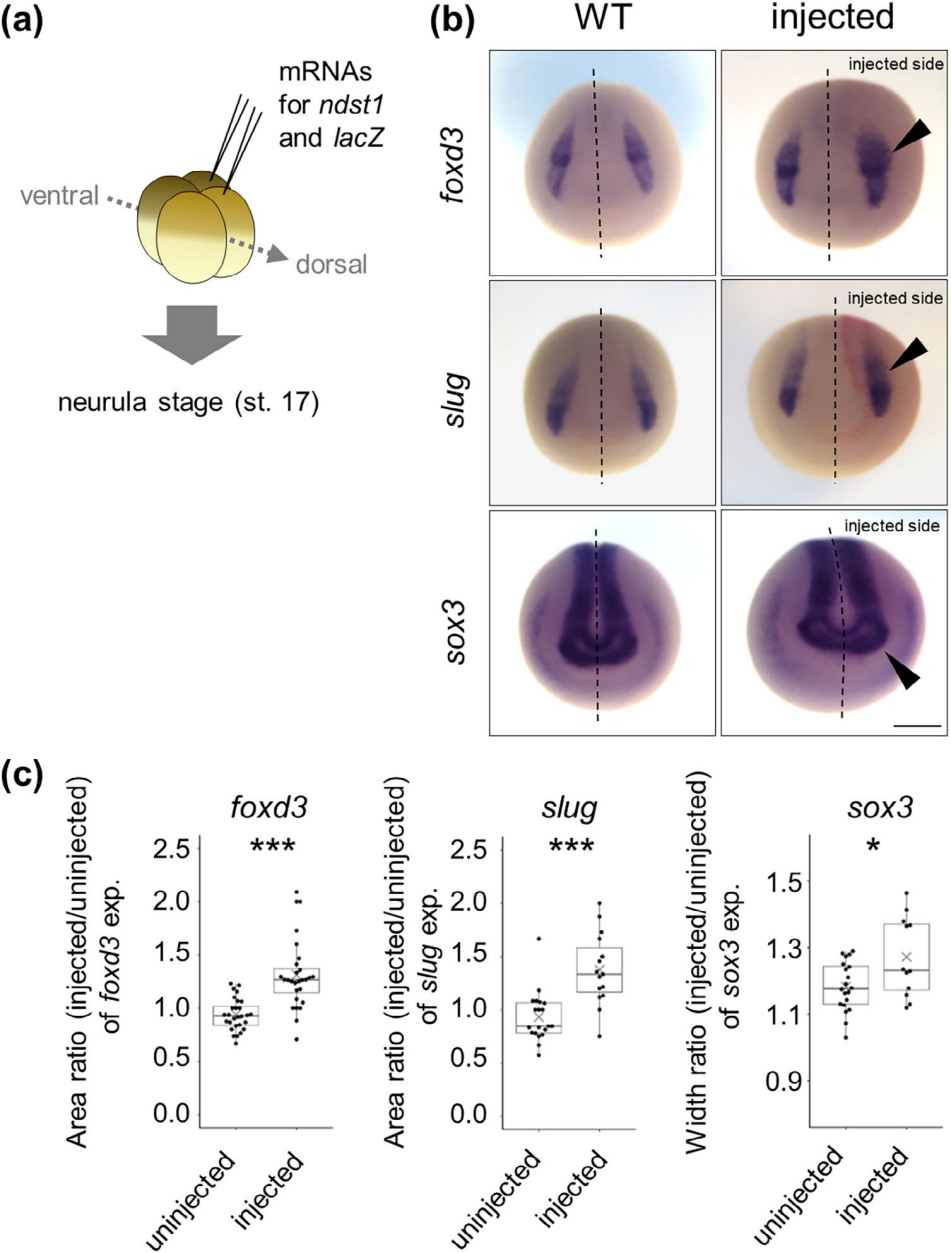
Overexpression of *ndst1* expands the neural crest region (a) Schematic view of the injection. 25 pg *ndst1.L* mRNA with a tracer, 100 pg *lacZ* mRNA, were injected into two blastomeres at the 4‐cell stage (slightly lateral from the midline as indicated), and the specimens were fixed at the neurula stage (st. 17). The light brown cells are the dorsal blastomere. The dark ones are the ventral blastomere. (b and c) Expression pattern of *foxd3*, *slug*, and *sox3* in *ndst1*‐overexpressed embryos. *lacZ* injected cells (a tracer) are colored red (“injected side” in the figures). *ndst1* overexpression expands the neuroectodermal‐gene expressed region as quantified in (c). Arrowheads indicate the expanded region. Dashed line indicates the midline of the embryo. Differences in expression areas were quantified by their area or width from the midline for the injected and uninjected sides. Statistical significance was analyzed by Student's *t*‐test (*p* = 1.1 × 10^−6^ [*foxd3*; *n* = 28 (uninjected) vs. 27 (injected)], 6.1 × 10^−5^ [*slug*; *n* = 20 (uninjected) vs. 15 (injected)], 0.014 [*sox3*; *n* = 20 (uninjected) vs. 12 (injected)]). *p < 0.05, ***p < 0.005. Scale bar = 500 μm.

Because *Xenopus laevis* is an allotetraploid species with two homeologous chromosomes, *L* and *S*, it has two *ndst1* genes, *ndst1.L* and *ndst1.S* (Session et al., 2016). During embryonic stages, the *L* gene is highly expressed (Michiue et al., 2017). To investigate role of *ndst1* during *Xenopus* early development, we injected *ndst1.L* mRNA into two blastomeres of the lateral region of the animal pole with a tracer *lacZ* mRNA at the 4‐cell stage (Figure 2a). Expression of NC marker genes, *slug* and *foxd3*, was expanded to the non‐neuroectodermal region on the injected side (Figure 2b,c). The anterior region of expression *of sox3*, an NP marker gene, was also expanded laterally. These expansions of expressing regions of *foxd3*, *slug*, and *sox3* in overexpressing embryos were statistically significant (Figure 2c), suggesting that *ndst1* expression regulates neuroectodermal patterning.

**FIGURE 3:**
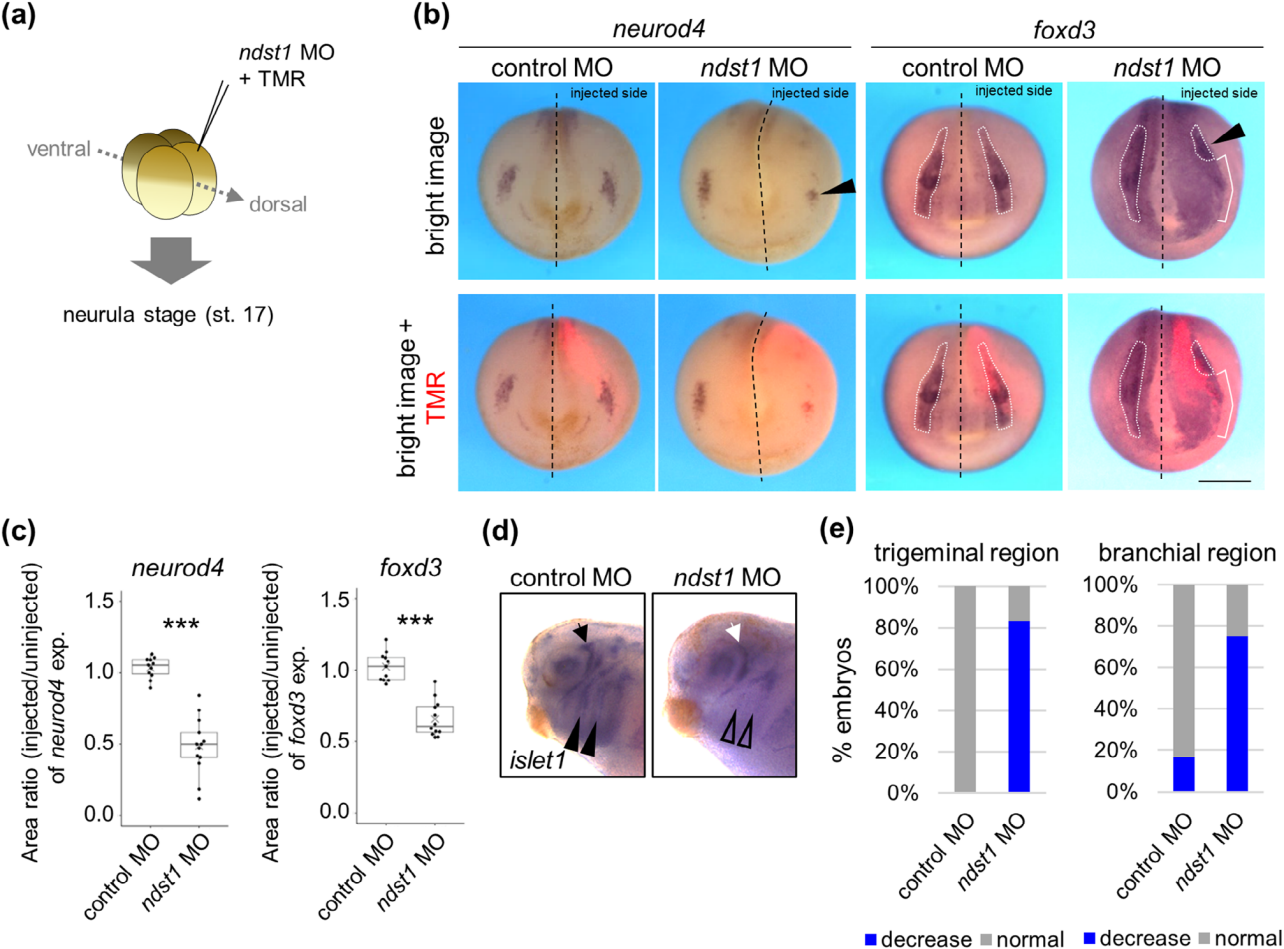
Knockdown of *ndst1* reduces the anterior neural crest region. (a) Schematic view of the injection. 2.5 pmol of *ndst1* morpholino antisense oligos with a tracer, tetramethylrhodamine (TMR), were injected into dorsal blastomere (dorsal and ventral blastomeres, slightly lateral from the midline as indicated), and specimens were fixed at the neurula stage (st. 17). (b and c) Expression pattern of *foxd3* and *neurod4*. TMR injected cells are colored red (the right side in the figures). This morpholino injection reduced the expressed region of both genes (arrowhead) as quantified in (c). *p* = 1.8 × 10^−8^ (*neurod4*; *n* = 11 (control morpholino antisense oligonucleotide [MO]) vs. 13 (*ndst1* MO)), 1.9 × 10^−7^ (*foxd3*; *n* = 11 (control MO) vs. 12 (*ndst1* MO)) (Student's *t*‐test). (d and e) *islet1* expression pattern at the tailbud stage (st. 32) in the morphants. *ndst1* knockdown reduces *islet1* expression in the trigeminal region (arrow), and the branchial region (arrowheads) as quantified in (e) (Fisher's exact test, *p* = 6.3 × 10^−5^ (trigeminal ganglia), 1.2 × 10^−2^ (cranial ganglia); *n* = 12 each). ***p < 0.005. Scale bar = 500 μm.

Similarities in expression patterns of *ndst1*, *neurod4*, and *islet1* suggest that *ndst1* functions in differentiation of the trigeminal nerve, which is derived from PPE. To examine this, we knocked down *ndst1* homeologs using a morpholino antisense oligonucleotide (MO), as previously reported (Mii et al., 2017). We injected *ndst1* MOs with a tracer, tetramethylrhodamine (TMR), into the slightly lateral region of the animal pole of a dorsal blastomere at the 4‐cell stage (Figure 3a). As a result, expression of a trigeminal marker gene, *neurod4*, decreased at the neurula stage (Figure 3b,c). In addition, anterior expression of an NC marker, *foxd3*, decreased (Figure 3b,c) and an epidermal marker gene, *krt12.4*, shrank laterally (Figure S2A,C). Although we wondered whether neighboring tissue would expand, the region expressing the NP marker, *sox3*, was not substantially altered (Figure S2A,B). Consistent with the defect in the trigeminal region at the neurula stage, *ndst1* further reduced expression of *islet1* in the trigeminal and branchial regions at the tailbud stage (Figure 3d,e). These results indicate that Ndst1 is essential at least for differentiation of the prospective trigeminal region and NC. In addition, *ndst1* knockdown may enhance differentiation of the neural/epidermal border other than NP, the trigeminal region, or NC because expression of all genes analyzed in this study was either reduced or unchanged.

The region affected by *ndst1* knockdown, the cranial region including the trigeminal region and NC, raises the possibility that acquisition of the *ndst* gene contributed to jaw evolution by shaping the morphogen gradient, including Wnt, because jaws are principally derived from the cranial region, which is a driving force of vertebrate evolution (Depew et al., 2002; Shigetani et al., 2002). At least, this gene, which has an N‐deacetylase/N‐sulfotransferase domain, is conserved not only in jawed vertebrates (gnathostomes), but also in *Ciona intestinalis*, *C. elegans*, *Drosophila*, cnidarians, and *Trichoplax*, suggesting that the *Ndst* gene appeared in the common ancestor of all metazoans (Filipek‐Górniok et al., 2015) although at least in *C. elegans* and *Drosophila* the sulfation site in sugar chains differs from that of vertebrate NDST (Kusche‐Gullberg et al., 2012; Toyoda et al., 2000). To further investigate whether this gene is conserved in cyclostomes (jawless vertebrates), we constructed a phylogenetic tree of putative *Ndst* genes of cyclostomes (lampreys and hagfish) and jawed vertebrates (Figure S3). Because putative *Ndst* genes of cyclostomes formed a monophyletic group with *Ndst* genes of jawed vertebrates, the *Ndst* gene(s) is apparently conserved in cyclostomes. However, only because the *Ndst* gene(s) itself is conserved, it does not necessarily mean that its expression pattern is also conserved. To further address whether Ndst is involved in jaw evolution, its expression pattern in cyclostomes should be analyzed in future studies.

**FIGURE 4:**
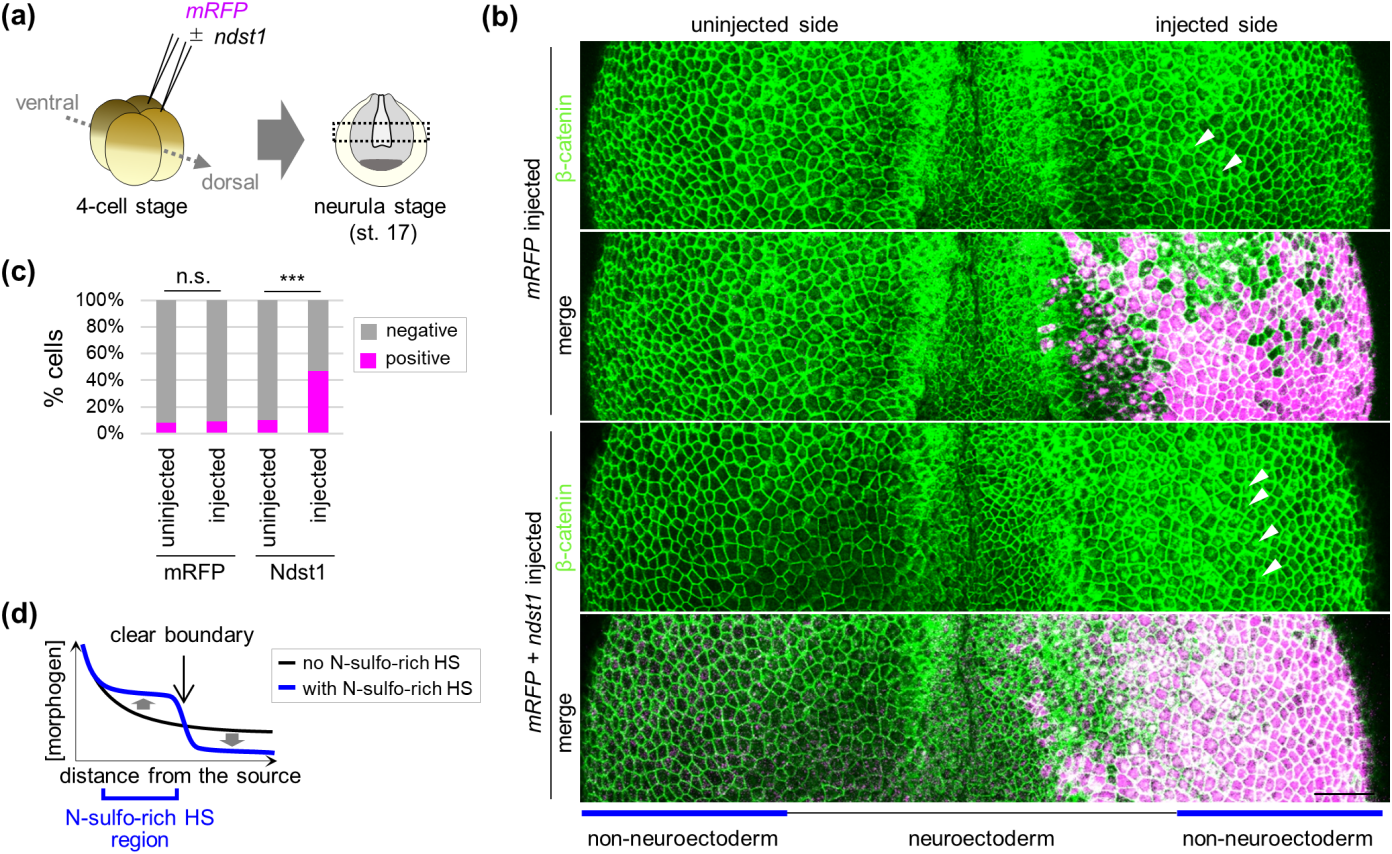
*ndst1* overexpression enhances Wnt activity at the neurula stage. (a) Schematic view of the injection. 400 pg *mRFP* mRNA with/without 25 pg *ndst1* mRNA was injected into two blastomeres at the 4‐cell stage as indicated. Specimens were fixed at the neurula stage (stage 17). (b and c) Number of cells positive for nuclear β‐catenin was increased by *ndst1*‐expression in the non‐neuroectodermal region. β‐catenin was visualized in the region that indicated by dotted box in (a) using immunohistochemistry against β‐catenin (green). Cells injected *mRFP* mRNA with/without *ndst1* mRNA are colored magenta. The neuroectodermal region is the center (black bar). The non‐neuroectodermal region is indicated by blue bars. Cells positive/negative for β‐catenin were counted in the non‐neuroectodermal region (uninjected side vs. injected side of *mRFP* injected or *mRFP* + *ndst1* injected embryos). Fisher's exact test, *p* = 0.84 (mRFP; n.s.; *n* = 172 [uninjected] vs. 143 [injected]), 1.4 × 10^−11^ (mRFP + Ndst1; ***; *n* = 131 [uninjected] vs. 130 [injected]). Scale bar = 100 μm. (d) Schematic view of N‐sulfo‐rich HS function on morphogen distribution.

Ndst1 enhances Wnt signaling at the gastrula stage (Mii et al., 2017) and region‐specific regulation of Wnt signaling is essential for neuroectodermal patterning (García‐Castro et al., 2002; Litsiou et al., 2005). Therefore, we wondered whether Ndst1 induces region‐specific enhancement of Wnt signaling during the neurula stage. To examine this, we injected *ndst1* mRNA with a tracer, *mRFP* mRNA into two blastomeres at the 4‐cell stage (Figure 4a), and analyzed nuclear localization of β‐catenin (an indicator of Wnt‐signal activation) using immunohistochemistry. Cells positive for nuclear β‐catenin were increased with *ndst1* expression, but not with *mRFP* expression (Figure 4b,c). This indicates that Ndst1 enhances Wnt signaling during neuroectodermal patterning.

Combined with the finding that *ndst1* expression enhances Wnt signaling (Figure 4b,c) and accumulates Wnt8a ligands (Mii et al., 2017), which are mainly secreted from the posterior region (Kiecker & Niehrs, 2001; Litsiou et al., 2005), *ndst1* knockdown is thought to reduce Wnt accumulation around Ndst1‐expressing cells, in the anterior NP and the trigeminal region (Figure 1), thereby reducing induction of the trigeminal region and NC. This is consistent with previous reports that Wnt is required for NC specification, but seemingly inconsistent with reports that Wnt signaling must be inhibited for specification of the precursor of the trigeminal region, PPE. However, Wnt signaling is also required when PPE is subdivided into smaller regions. Among the smaller regions of PPE derivatives, the trigeminal placode region requires a higher level of Wnt activation (Heisenberg et al., 2001; Park & Saint‐Jeannet, 2008; Watanabe et al., 2015). This can explain the PPE defect, a reduction of trigeminal formation, in *ndst1*‐knocked‐down embryos.

However, Ndst1‐deficient mice embryos show defects similar to mutants of the FGF and Shh pathways (Pallerla et al., 2007). In neuroectodermal patterning, FGF signaling is required for NP, NC, and PPE specification (Ahrens & Schlosser, 2005; Litsiou et al., 2005; Streit & Stern, 1999). Our finding that Ndst1 regulates Wnt signaling at the neurula stage does not rule out the possibility that it also regulates FGF signaling.

We also revealed that *ndst1* affects not only cells in which it is expressed, but also neighboring cells, including NC (Figure 3). Accumulation of Wnt ligand by Ndst1 enhances Wnt signaling near *ndst1*‐expressing cells but inhibits further Wnt spreading. This may broadly affect neuroectodermal patterning, not limited to *ndst1*‐expressing cells, as observed in *ndst1*‐knockdown embryos.

In general, to form a morphogen gradient, it is necessary to have a “sink” that removes morphogen from the extracellular space (Crick, 1970). Because N‐sulfo‐rich HS, which is the product of Ndst1, and is present more or less throughout embryos at the gastrula stage, is readily internalized from the cell membrane with morphogen, we thought that it not only accumulates morphogens but also serves as a morphogen “sink” (Mii et al., 2017). Additionally, the present data suggest that N‐sulfo‐rich HS localized in specific regions of embryos actively reduces the amount of morphogen in regions distal to the morphogen source, clearly defining boundaries of morphogen‐mediated signaling, rather than merely shaping gradients (Figure 4d). Thus, region‐specific removal of morphogen mediated by N‐sulfo‐rich HS may be essential to form complex gradients for proper patterning, including neuroectodermal differentiation in vertebrates.

## AUTHOR CONTRIBUTIONS

Takayoshi Yamamoto, Yuta Kambayashi, Kohei Tsukano, and Tatsuo Michiue conceived this project. Takayoshi Yamamoto, Yuta Kambayashi and Kohei Tsukano performed experiments and wrote the manuscript with help from Tatsuo Michiue.

## CONFLICT OF INTEREST STATEMENT

The authors declare no competing interests.

## Supporting information


**Figure S1.** *ndst1* expression pattern at early neurula stage in *Xenopus*. Anterior view of *ndst1* expression at stage 13 (dorsal to the top). Scale bar = 500 μm.

**Figure S2.**Knockdown of *ndst1* reduced the epidermal region, but not the neural plate region. (A) Schematic view of the injection. 2.5 pmol of *ndst1* morpholino antisense oligos with a tracer, TMR, were injected into the dorsal blastomere, and specimens were fixed at the neurula stage (st. 17). (B–C) Expression pattern of *sox3* and *krt12.4*. TMR injected cells are colored red (the right side in each figure). This morpholino injection reduced *krt12.4*‐expressed region (arrowhead; *n* = 25 [control MO], *n* = 26 [*ndst1* MO]), but not *sox3*‐expressed region (*n* = 29/30 [control MO], *n* = 28/30 [*ndst1* MO]). Differences in *krt12.4*‐expressed region were quantified by the width from the midline to the place where the expression is visible, as shown by double‐headed arrows in the figures. ***p = 4.25 × 10^−5^ (Student's *t*‐test). Scale bar = 500 μm.

**Figure S3.** Phylogenetic tree of *ndst* genes. The tree was constructed with coding sequences of *ndst* genes using the maximum likelihood method. As the outgroup, we used coding sequences of *hs3st genes* of *Mus musculus*, *Gallus gallus*, and *X. tropicalis* because these genes showed the lowest E values other than *ndst* genes. Each branch is labeled with the species name, gene ID, and gene name, in that order. However, if the gene was not annotated, the gene name is not shown in the figure. Green lines indicate genes of cyclostomes. Blue lines indicate cephalochordates. Magenta lines indicate echinoderms.
