# Supplementary figures and images for: Ndst1, a heparan sulfate modification enzyme, regulates neuroectodermal patterning by enhancing Wnt signaling in Xenopus
Source: Dev Growth Differ. 2023 Mar 3;65(3):153–60. doi: 10.1111/dgd.12843 (PMC11520968; doi:10.1111/dgd.12843)

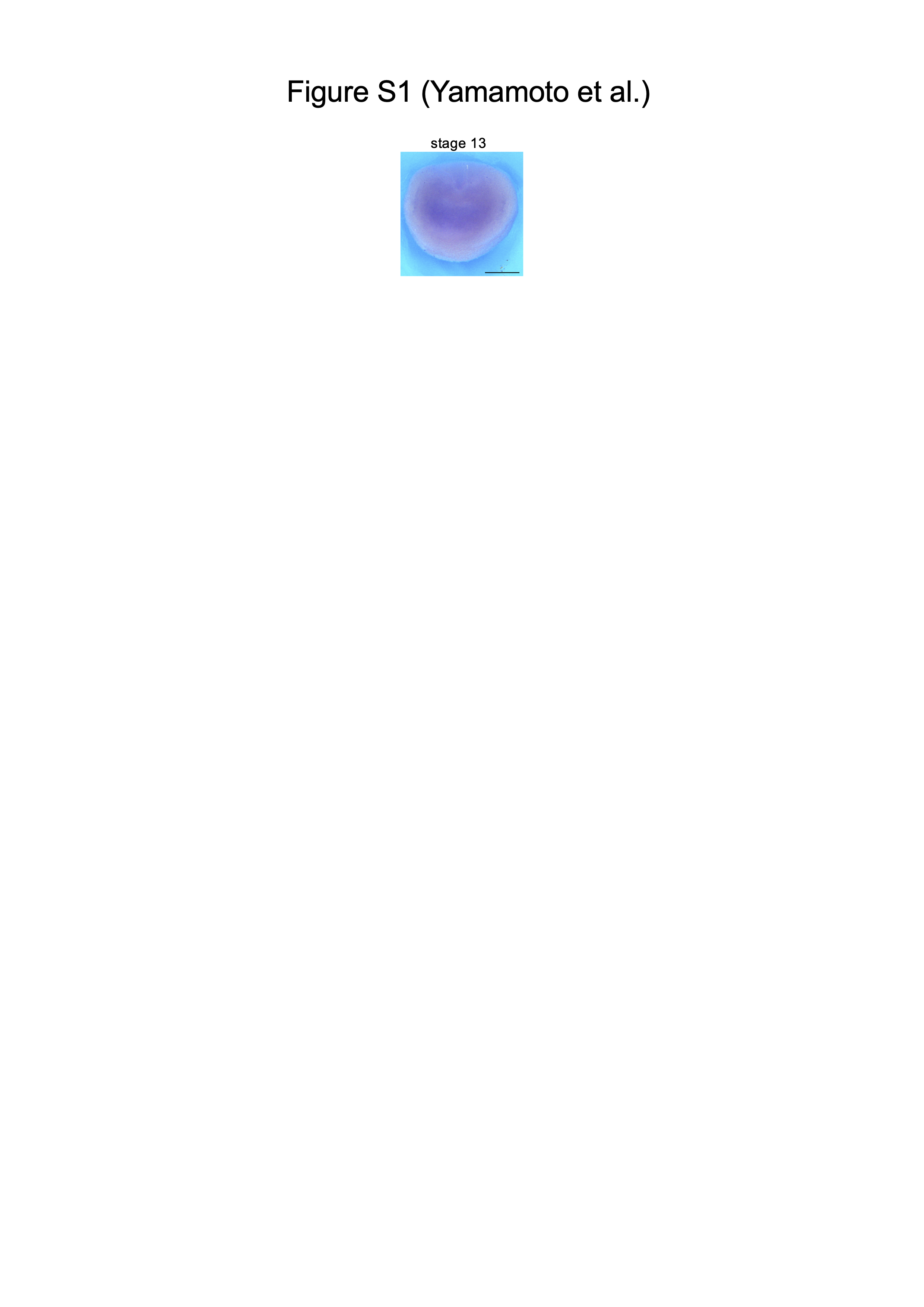

Supplement: Supplementary file 1 — Figure S1. ndst1 expression pattern at early neurula stage in Xenopus. Anterior view of ndst1 expression at stage 13 (dorsal to the top). Scale bar = 500 μm. [file DGD-65-153-s001.tiff]

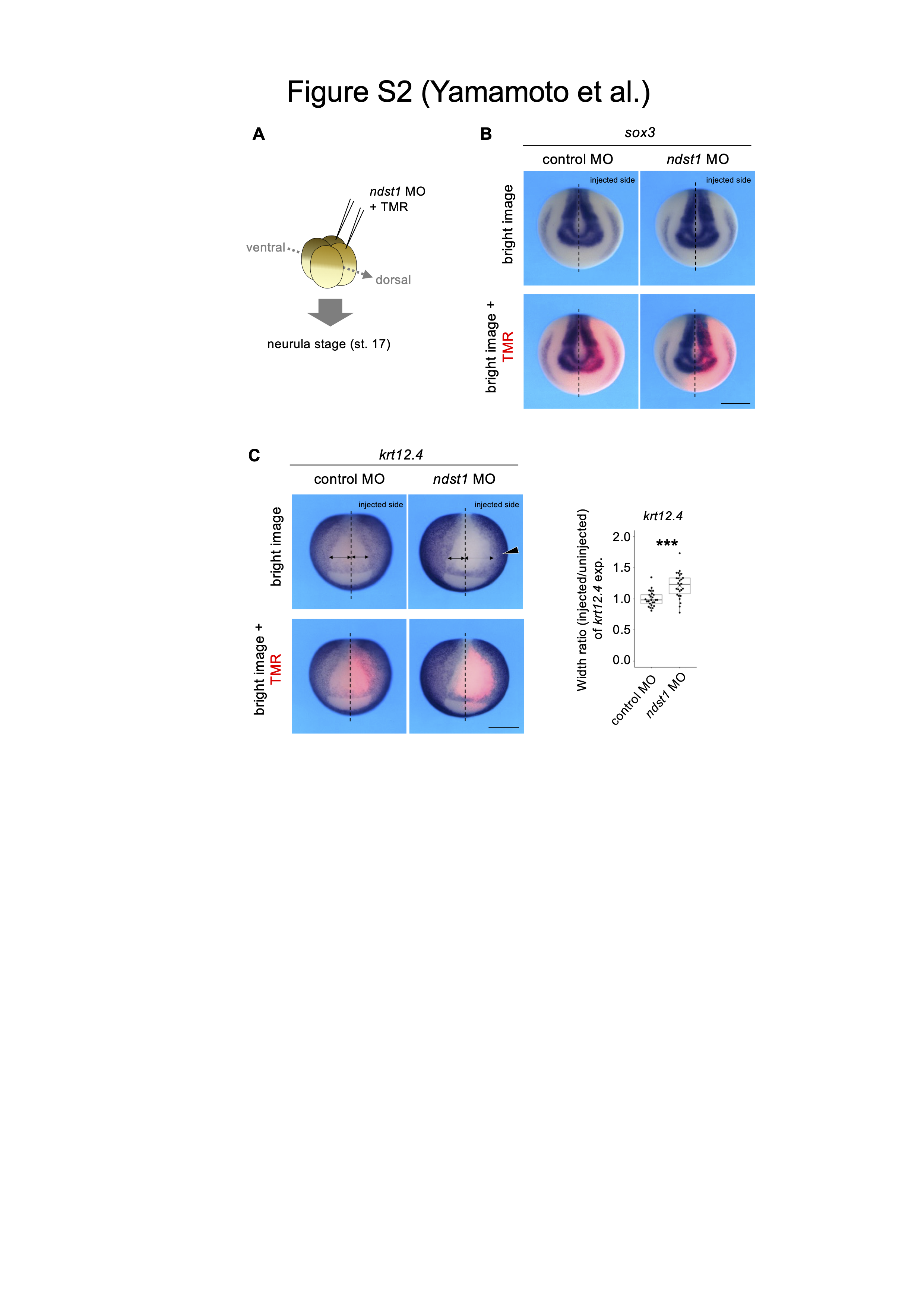

Supplement: Supplementary file 2 — Figure S2.Knockdown of ndst1 reduced the epidermal region, but not the neural plate region. (A) Schematic view of the injection. 2.5 pmol of ndst1 morpholino antisense oligos with a tracer, TMR, were injected into the dorsal blastomere, and specimens were fixed at the neurula stage (st. 17). (B–C) Expression pattern of sox3 and krt12.4. TMR injected cells are colored red (the right side in each figure). This morpholino injection reduced krt12.4‐expressed region (arrowhead; n = 25 [control MO], n = 26 [ndst1 MO]), but not sox3‐expressed region (n = 29/30 [control MO], n = 28/30 [ndst1 MO]). Differences in krt12.4‐expressed region were quantified by the width from the midline to the place where the expression is visible, as shown by double‐headed arrows in the figures. ***p = 4.25 × 10^−5^ (Student's t‐test). Scale bar = 500 μm. [file DGD-65-153-s002.tiff]

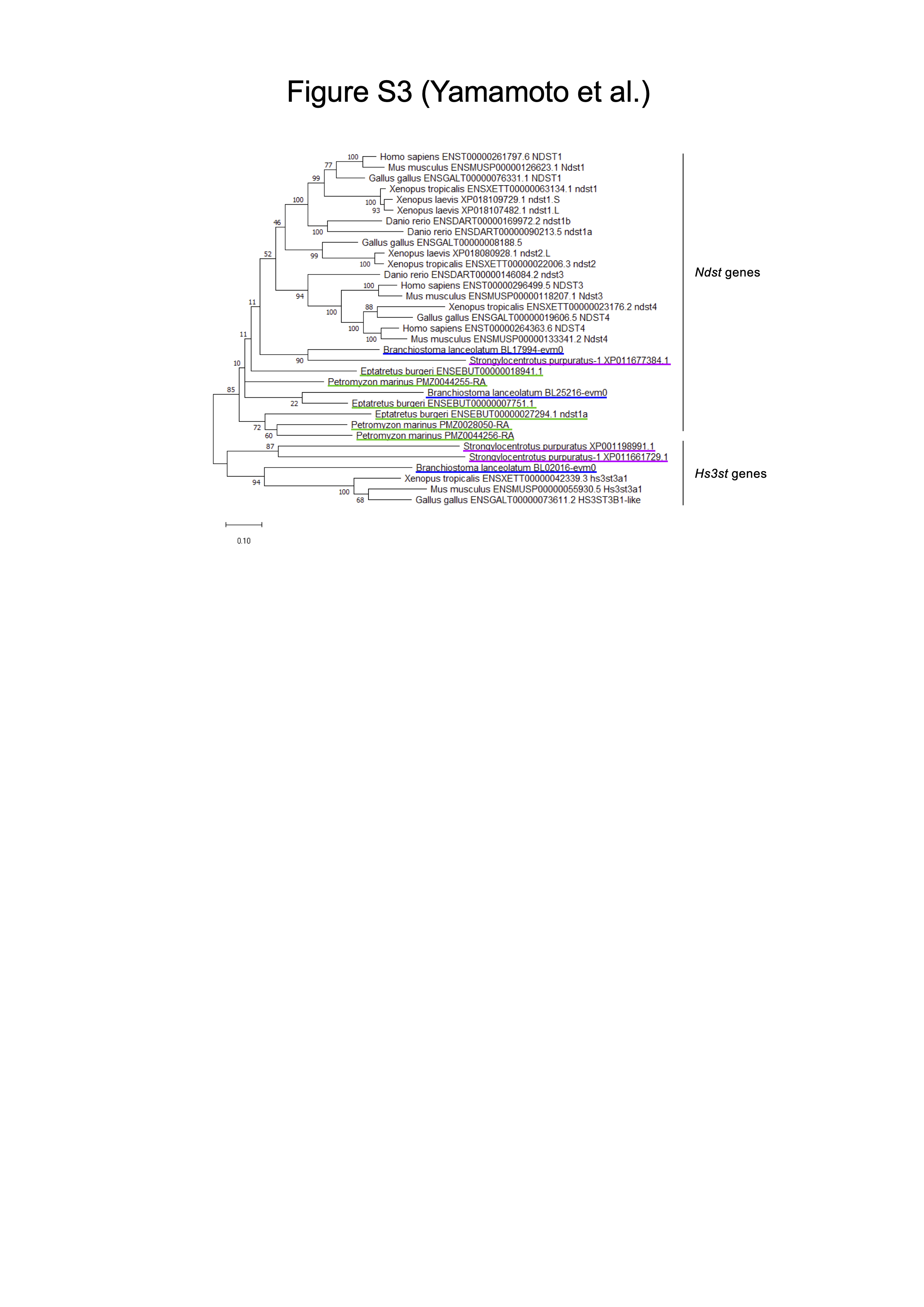

Supplement: Supplementary file 3 — Figure S3. Phylogenetic tree of ndst genes. The tree was constructed with coding sequences of ndst genes using the maximum likelihood method. As the outgroup, we used coding sequences of hs3st genes of Mus musculus, Gallus gallus, and X. tropicalis because these genes showed the lowest E values other than ndst genes. Each branch is labeled with the species name, gene ID, and gene name, in that order. However, if the gene was not annotated, the gene name is not shown in the figure. Green lines indicate genes of cyclostomes. Blue lines indicate cephalochordates. Magenta lines indicate echinoderms. [file DGD-65-153-s003.tiff]
